# Supplementary material for: ProteinShader: illustrative rendering of macromolecules
Source: BMC Struct Biol. 2009 Mar 30;9:19. doi: 10.1186/1472-6807-9-19 (PMC2672931; doi:10.1186/1472-6807-9-19)
Supplement: Additional file 1 — ProteinShader program without source code. This compressed file contains the complete ProteinShader program including associated libraries, but no source code. A README.txt file gives an overview of the ProteinShader distribution, and the index.html file in the help subdirectory has directions on getting started with the program as well as a set of tutorials. [file 1472-6807-9-19-S1.zip › ProteinShader-beta-0_9_4-binary/help/api/org/proteinshader/gui/listeners/menubar/VisibilityMenuListenerFactory.html]

VisibilityMenuListenerFactory (ProteinShader API)


|  |  |  |
| --- | --- | --- |
| **Overview** \| **Package** \| **Class** \| **Use** \| **Tree** \| **Deprecated** \| **Index** \| **Help** | |  |
| **PREV CLASS**   NEXT CLASS | **FRAMES**    **NO FRAMES**     **All Classes** |  |
| SUMMARY: NESTED \| FIELD \| CONSTR \| METHOD | DETAIL: FIELD \| CONSTR \| METHOD |  |


---


## org.proteinshader.gui.listeners.menubar Class VisibilityMenuListenerFactory

```
java.lang.Object
  org.proteinshader.gui.listeners.menubar.VisibilityMenuListenerFactory
```

---

``` public class VisibilityMenuListenerFactory extends Object ```

Creates all event listeners needed by the VisibilityMenu of the MainMenuBar.

---

**Constructor Summary**

`VisibilityMenuListenerFactory(Mediator mediator)`
  
          Constructs a VisibilityMenuListenerFactory.


| **Method Summary** | |
| --- | --- |
| `ActionListener` | `createAminoAcidsCheckBoxListener()`             Creates an action listener for the 'Amino Acids' check box menu item. |
| `ActionListener` | `createHeterogensCheckBoxListener()`             Creates an action listener for the 'Heterogens' check box menu item. |
| `ActionListener` | `createWatersCheckBoxListener()`             Creates an action listener for the 'Waters' check box menu item. |

**Methods inherited from class java.lang.Object**

`clone, equals, finalize, getClass, hashCode, notify, notifyAll, toString, wait, wait, wait`

**Constructor Detail**

### VisibilityMenuListenerFactory

```
public VisibilityMenuListenerFactory(Mediator mediator)
```

:   Constructs a VisibilityMenuListenerFactory.

    **Parameters:**: `mediator` - the centralized Mediator object that listeners call on to accomplish their task.


**Method Detail**

### createAminoAcidsCheckBoxListener

```
public ActionListener createAminoAcidsCheckBoxListener()
```

:   Creates an action listener for the 'Amino Acids' check box menu
    item. When the listener is called, it will tell the Mediator to
    show the AminoAcids if the check box is selected, or to hide them
    if the check box is deselected.

    :   **Returns:**: The action listener.

---


### createHeterogensCheckBoxListener

```
public ActionListener createHeterogensCheckBoxListener()
```

:   Creates an action listener for the 'Heterogens' check box menu
    item. When the listener is called, it will tell the Mediator to
    show the Heterogens if the check box is selected, or to hide them
    if the check box is deselected.

    :   **Returns:**: The action listener.

---


### createWatersCheckBoxListener

```
public ActionListener createWatersCheckBoxListener()
```

:   Creates an action listener for the 'Waters' check box menu item.
    When the listener is called, it will tell the Mediator to show the
    Waters if the check box is selected, or to hide them if the check
    box is deselected.

    :   **Returns:**: The action listener.


---


|  |  |  |
| --- | --- | --- |
| **Overview** \| **Package** \| **Class** \| **Use** \| **Tree** \| **Deprecated** \| **Index** \| **Help** | |  |
| **PREV CLASS**   NEXT CLASS | **FRAMES**    **NO FRAMES**     **All Classes** |  |
| SUMMARY: NESTED \| FIELD \| CONSTR \| METHOD | DETAIL: FIELD \| CONSTR \| METHOD |  |


---

# *Copyright © 2007-2008*
